# Correlation Between Diabetic Cognitive Impairment and Diabetic Retinopathy in Patients With T2DM by ^1^H-MRS

**DOI:** 10.3389/fneur.2019.01068

**Published:** 2019-11-12

**Authors:** Xuefang Lu, Wei Gong, Zhi Wen, Lanhua Hu, Zhoufeng Peng, Yunfei Zha

**Affiliations:** Department of Radiology, Renmin Hospital, Wuhan, China

**Keywords:** T2DM, diabetic brain damage, diabetic retinopathy, hippocampus, ^1^H-MRS, MoCA

## Abstract

**Objective:** To explore the correlation between diabetic cognitive impairment (DCI) and diabetic retinopathy (DR) through examining the cognitive function and the metabolism of the cerebrum in Type 2 diabetes mellitus (T2DM) by ^1^H-MRS.

**Methods:** Fifty-three patients with T2DM were enrolled for this study. According to the fundus examination, the patients were divided into the DR group (*n* = 26) and the T2DM without DR group (T2DM group, *n* = 27). Thirty healthy adults were selected as a control group (HC group, *n* = 30). Cognitive function was measured by Montreal Cognitive Assessment (MoCA). The peak areas of N-acetylaspartate (NAA), Cho-line (Cho), Creatine (Cr), and Myo-inositol (mI) as well as their ratios were detected by proton magnetic resonance spectroscopy (^1^H-MRS). The difference analysis between the three groups was performed by one-way ANOVA. When *p* < 0.05, LSD-t was applied. A partial correlation analysis (with age as a covariate) was used to analyze the correlation between metabolites in the DR group and MoCA scores. Among all T2DM patients, Chi-square test age, gender, education level, BMI, SBP, DBP, FPG, HbA1c, TC, TG, HDL-C, LDL-C, DR, and DCI correlation were measured. Differences were statistically significant while *P* < 0.05.

**Results:** 1. The scores of MoCA in the DR group or in the T2DM group were significantly less than those in the HC group (*F* = 3.54, *P* < 0.05), and the scores of MoCA in the DR group were significantly less than those in the other groups (*F* = 3.61, *P* < 0.05). 2. There were significant differences for NAA in the bilateral hippocampus in DR patients, T2DM patients, and healthy controls (*P* < 0.05). 3. The NAA/Cr was significantly positively correlated with the score of MoCA in DR patients' left hippocampus (*r* = 0.781, *P* < 0.01). 4. Chi-square analysis found that there was a correlation between DR and DCI (*x*^2^ = 4.6, df = 1, *p* = 0.032, plt: 0.05). There was no correlation between other influencing factors and DCI (*P* > 0.05).

**Conclusion:** DCI is closely correlated with the DR in patients with T2DM. Hippocampal brain metabolism may have some changes in two sides of NAA in patients with DR, ^1^H-MRS may provide effective imaging strategies and methods for the early diagnosis of brain damage and quantitative assessment cognitive function in T2DM.

Type 2 diabetes mellitus (T2DM) is a comprehensive metabolic disease caused by insufficient insulin secretion or insulin resistance and is associated with a variety of systemic complications (1). The incidence of T2DM in China has reached 9.7% (2). T2DM has similar pathological changes with Alzheimer's disease (AD), namely axonal degeneration, neuronal loss, and extensive fibrosis of the meninges, while the hippocampus undergoes one of the early changes in T2DM brain structure (3), with varying degrees of cognitive impairment. The Montreal Cognitive Assessment Scale (MoCA) is able to assess cognitive impairment, including naming abilities, visual space, and executive ability. Studies have shown that diabetic patients with diabetic cognitive impairment (DCI) have a higher risk of cardiovascular accidents and death than diabetic patients without cognitive impairment (4). Studies (5) found that diabetic retinopathy (DR) may be closely related to DCI and provide indirect evidence of cognitive dysfunction in diabetes, but its clinical symptoms are diverse and unstable, and there is still a lack of prevention. The gold standard for prevention is often easily underestimated or ignored. ^1^H-MRS (proton magnetic resonance spectroscopy) is a powerful tool for early detection and quantitative assessment of brain microstructural and functional changes in T2DM patients and brings the unique advantages of virtual biopsy. The correlation between cognitive impairment and DR is explored through examining the cognitive function and the metabolism of the cerebrum in T2DM by ^1^H-MRS and MoCA.

## Objects and Methods

### Objects

This study was approved by the patients and the ethics committee. Subjects were recruited from January 2016 to December 2018 in our hospital, according to the T2DM diagnostic criteria promulgated by the American Diabetes Association (ADA) in 2010. To avoid as much as possible the course of diabetes, the drugs taken, the way of treatment and the complications of diabetes and other neurological diseases. The course of diabetes was more than 15 years but <30 years. The patients were not using insulin and had standardized oral medication. There were differences in diabetic retinopathy, and none had any other diabetic complications or microangiopathies. Fundus examination was used to diagnose the presence or absence of DR lesions, microaneurysms, and/or small hemorrhage as it is the earliest and most accurate characteristics of DR lesions, and 53 patients with T2DM were enrolled for this study. According to the fundus examination, the patients were divided into the DR group (*n* = 26) and the T2DM without DR group (T2DM group, *n* = 27). HC (healthy control group) group inclusion criteria were as follows: no abnormal glucose metabolism, and other conditions were matched with DR and T2DM. Thirty healthy adults were selected as the control group (HC group, *n* = 30).

Exclusion criteria were organized into three groups: (1) patients who could not complete the MoCA test; (2) patients with a history of mental and neurological diseases; (3) those with organic diseases of the nervous system; (4) patients who were dependent on smoking, alcoholism and psychotropic substances; (5) impaired glucose tolerance or fasting glucose, and ketoacidosis; and (6) patients with contraindications with MRI. There were differences in diabetic retinopathy, they all had no other diabetic complications, and there were no significant differences in gender, age, education, height, and weight (*P* > 0.05); there were statistical differences among three groups of GLU and HbA1c (*P* < 0.05) (Table 1).

**Table 1:** Comparison of the general conditions of the three groups of subjects.

**Project**	**DR group**	**T2DM group**	**HC group**	***t***	***P***
Gender (M/F)	26 (14/12)	27 (10/17)	30 (17/13)	5.163	0.076
Age (y)	61.19 ± 8.41	61.04 ± 7.54	59.81 ± 3.68	0.249	0.780
Education (y)	9.50 ± 1.90	10.54 ± 3.40	11.12 ± 2.70	1.587	0.212
BMI (kg/m^2^)	23.94 ± 2.52	23.69 ± 3.04	23.37 ± 3.64	0.169	0.845
SBP/mmHg	160.24 ± 9.43	160.01 ± 7.74	150.18 ± 9.81	8.291	0.801
DBP/mmHg	96.19 ± 8.54	96.22 ± 8.75	90.01 ± 9.85	7.342	0.786
FPG (mmol/L)	9.23 ± 0.46	7.18 ± 0.82	5.13 ± 0.37	11.203	0.000^*^
HbA1c (%)	8.98 ± 2.24	7.98 ± 0.89	5.31 ± 0.40	3.670	0.000^*^
TC (mmol/L)	6.32 ± 1.07	5.37 ± 1.16	4.31 ± 1.12	1.037	0.000^*^
TG (mmol/L)	1.98 ± 0.97	1.92 ± 1.09	1.07 ± 0.79	0.901	0.000^*^
HDL-C (mmol/L)	0.89 ± 0.13	1.02 ± 0.46	1.38 ± 0.72	0.870	0.000^*^
LDL-C (mmol/L)	3.81 ± 0.71	3.76 ± 0.57	3.51 ± 0.45	1.015	0.000^*^

* *There were statistical differences among three groups in FPG, HbA1c, TC, TG, HDL-C, and LDL-C (P <0.05)*.

*There were no significant differences among three groups in gender, age, education, BMI, SBP, and DBP (P > 0.05)*.

Cognitive function was measured by MoCA. The peak areas of N-acetylaspartate (NAA), Cho-line (Cho), Creatine (Cr), and Myo-inositol (mI), and their ratios, were detected by ^1^H-MRS.

### Methods

#### Cognitive Function Test and Fundus Examination

The MoCA assessment was performed by a neurology professional and an ophthalmologist performed the fundus examination. This study used an internationally recognized version developed in November 2004. The scale was developed by Nasreddine et al. (6) with reference to the Mini-mental State Examination and based on clinical experience. It is an assessment tool for rapid screening of cognitive dysfunction. It includes 11 inspection projects in 8 cognitive areas, including concentration, executive function, memory, language, visual structural skills, abstract thinking, calculation, and positioning. It possesses high sensitivity, covers important cognitive areas, has a short test time and is suitable for clinical settings.

$$\text{n}=\;\frac{2\bar{p}\bar{q}{\left({\text{Z}}_{\alpha }\;+\;{\text{Z}}_{\beta }\right)}^{2}}{{\text{(p1 - p2)}}^{\text{2}}}$$

Here, *n* indicates the number of samples, and the table shows that Z_α_, Z_β_is 1.96 and 1.28, respectively. p_1_ and p_2_ represent the prevalence of the intervention group and the control group, with $\bar{p}$ representing the average of p_1_ and p_2_, and $\bar{q}$ representing the average of (1–p_1_) and (1–p_2_).

The incidence of T2DM in China is as high as 9.7% (2). According to the above formula, we get *n* is equal to 6.38, and the total number of diabetic patients enrolled is 53, so the sample size is reasonable and the result is credible.

#### Equipment and Methods

This study used a GE (Discovery 750w) 3.0T superconducting magnetic resonance imaging scanner and matching head coil (8 channels), and quality testing was employed before each scan to ensure the stability of the machine signal. All objects underwent a T2WI scan in advance, and two professional imaging physicians evaluated the images to rule out brain lesions. A high-resolution 3D-T1 multi-planar image was obtained by 3D-T1 MPRAGE for subsequent scan positioning. The following conditions were present: hippocampus ^1^H-MRS, STEAM (Stimulated Echo Acquisition Method) mode, point probe Probe-SV35 sequence scan (7), TR 1700 ms, TE 30 ms, FA 90°, ST 15 mm, FOV 16 cm × 16 cm, MS 320 × 320, VS 10 mm × 10 mm × 15 mm. The scan time is usually 6 min 53 s. Coastal horse long axis, according to the axial ROI, supplemented by sagittal, oblique coronal plane, try to avoid air cavity, skull, cerebrospinal fluid, and fat. The bilateral ROI was the same and the whole hippocampus was included as much as possible. The scan was manually corrected and the value was read at the workstation.

GE (Discovery 750w) 3.0T workstation spectroscopy software was used for phase and baseline calibration of the measured line, signal averaging, metabolite identification and spectral line fitting, automatic reading of the bilateral hippocampus, the area under the peak curve of NAA, Cho, Cr, and mI and to calculate the ratio of NAA/Cr, Cho/Cr, and mI/Cr.

#### Statistical Method

All data were tested and conformed to a normal distribution by SPSS21.0 software package. The statistical analysis of the analytes obtained by ^1^H-MRS was performed. The data are represented as the mean ± standard deviation. The difference analysis between the three groups was performed by one-way ANOVA. When *p* < 0.05, LSD-t was applied. A partial correlation analysis (with age as a covariate) was used to analyze the correlation between metabolites in the DR group and MoCA scores. Among all T2DM patients, Chi-square test was used on age, gender, education level, BMI, SBP, DBP, FPG, HbA1c, TC, TG, HDL-C, LDL-C, DR, and DCI correlation. Differences were statistically significant while *P* < 0.05.

## Results

### MoCA

One-way ANOVA statistical analysis showed that the MoCA scores in the DR group were significantly less than those in the other groups (*P* < 0.05), and the MoCA scores in the T2DM group were significantly less than those in the HC group (*P* < 0.05).

### Comparison of Bilateral Hippocampal ^1^H-MRS Detection Values Between DR Group, T2DM Group, and HC Group

**Figure 1:**
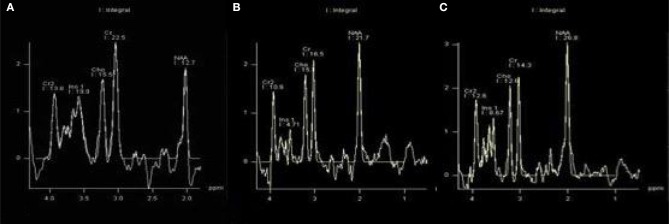
There were statistically significant differences in NAA on bilateral hippocampus. **(A)** DR group, **(B)** T2DM group, **(C)** HC group.

**Figure 2:**
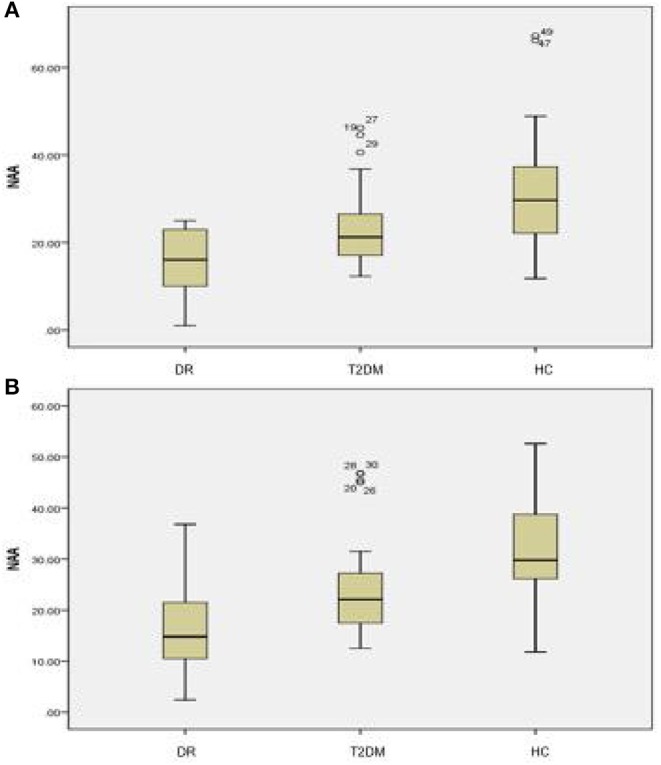
There were statistically significant differences in NAA on the bilateral hippocampus among the DR group, the T2DM group and the HC group. **(A)** Left hippocampal, **(B)** right hippocampal.

There were statistically significant differences in NAA on the bilateral hippocampus between the DR group, the T2DM group and the HC group (*F*_left_ = 10.052, *p* < 0.05; *F*_right_ = 10.316, *p* < 0.05). Further examination with LSD-t showed that the DR group was significantly lower than the T2DM and HC groups (*p* < 0.05) (Figures 1, 2, Tables 2, 3); the differences of the other indicators were not statistically significant (*P* > 0.05).

**Table 2:** Multiple comparisons of left hippocampal NAA.

**Group**		**Mean**	**SD**	***p***	**95% confidence interval**	
					**Lower limit**	**Upper limit**
DR	T2DM	-8.12260^*^	3.56952	0.026^*^	−15.2494	−0.9958
	HC	-15.29449^*^	3.54425	0.000^*^	−22.3708	−8.2182
T2DM	DR	8.12260^*^	3.56952	0.026^*^	0.9958	15.2494
	HC	-7.17189^*^	3.08675	0.023^*^	−13.3348	−1.0090
HC	DR	15.29449^*^	3.54425	0.000^*^	8.2182	22.3708
	T2DM	7.17189^*^	3.08675	0.023^*^	1.0090	13.3348

* *The left hippocampal NAA were compared between any two groups, p <0.05, the difference was statistically significant. T2DM, Type 2 diabetes mellitus; DR, diabetic retinopathy; NAA, Nacetylaspartate*.

**Table 3:** Multiple comparisons of right hippocampal NAA.

**Group**		**Mean**	**SD**	***p***	**95% confidence interval**	
					**Lower limit**	**Upper limit**
DR	T2DM	−7.24610^*^	3.06174	0.021^*^	−13.3557	−1.1365
	HC	−13.85166^*^	3.06174	0.000^*^	−19.9613	−7.7421
T2DM	DR	7.24610^*^	3.06174	0.021^*^	1.1365	13.3557
	HC	−6.60556^*^	2.69142	0.017^*^	−11.9762	−1.2349
HC	DR	13.85166^*^	3.06174	0.000^*^	7.7421	19.9613
	T2DM	6.60556^*^	2.69142	0.017^*^	1.2349	11.9762

* *The right hippocampal NAA was compared between any two groups, p <0.05, the difference was statistically significant. T2DM, Type 2 diabetes mellitus; DR, diabetic retinopathy; NAA, Nacetylaspartate*.

### Correlation Between ^1^H-MRS and MoCA Scores in DR Group

The NAA/Cr is significantly positively correlated with the MoCA score in DR patients on the left hippocampus (*r* = 0.774, *P* < 0.01). There were no correlations among the other detection values of the two hippocampus detection values and the MoCA scores (*P* > 0.05) (Table 4).

**Table 4:** Correlation between the detection values of bilateral hippocampus and MoCA score in DR group.

**Project**	**NAA**	**Cr**	**Cho**	**mI**	**NAA/Cr**	**Cho/Cr**	**mI/Cr**
*r*_left_	0.312	−0.523	−0.411	−0.046	0.684	0.441	0.292
*p*_left_	>0.05	>0.05	>0.05	>0.05	<0.01^※※^	>0.05	>0.05
*r*_right_	−0.271	−0.299	−0.121	0.312	0.041	0.402	0.476
*p*_right_	>0.05	>0.05	>0.05	>0.05	>0.05	>0.05	>0.05

※※ *The ratio of NAA/Cr in the left hippocampal of DR patients was positively correlated with MoCA score, p <0.01, and the difference was statistically significant. NAA, Nacetylaspartate; Cr, Creatine; Cho, Cho-line; mI, myo-inositol*.

Among all T2DM patients, Chi-square tested age, gender, education level, BMI, SBP, DBP, FPG, HbA1c, TC, TG, HDL-C, LDL-C, DR, and DCI for correlation. Chi-square analysis found that there was a correlation between DR and DCI (*x*^2^ = 4.6, df = 1, *p* = 0.032, plt: 0.05). There was no correlation between other influencing factors and DCI (*P* > 0.05).

## Discussion

With changes in modern lifestyles, the incidence of T2DM is increasing year by year (8). The incidence of cognitive dysfunction caused by T2DM is 10.8–17.5% (9), and its occurrence is related to hippocampus and amygdala atrophy (10). In addition, T2DM not only causes metabolic disorders but also involves multiple systems. DR is one of the more common lesions, and studies have shown that diabetic retinopathy is closely related to DCI (11). The occurrence of these two diseases is parallel (12, 13): (1) mass accumulation of glycosylation end products; (2) the protein kinase C (PKC) pathway is activated at high glucose; (3) oxidative stress: there is an excessive amount of reactive oxygen species, resulting in vascular cell damage, diabetic brain damage, or DR; and (4) DR is also a neurovascular disease, which, like diabetic brain injury, can be specifically reflected by the neuronal marker NAA in the ^1^H-MRS.

The literature reports that there is early brain tissue damage in patients with diabetic retinopathy, and ^1^H-MRS can detect this change early (12, 13). In recent years, ^1^H-MRS has been reported in the diagnosis of cognitive impairment-related diseases, but there are few reports on the relationship between DCI caused by T2DM and DR. Based on the above problems, the research team—based on the previous animal experiments—explored the correlation between DCI and DR through examining the cognitive function and the metabolism of the cerebrum in T2DM by ^1^H-MRS.

Studies have shown that individual factors such as education level and age group have different degrees of impact on MoCA scores (14). In this study, because the subjects were matched as much as possible in addition to the diabetes itself, the credibility of DCI judgment was higher, and these objects mainly showed memory loss, which is similar to previous reports (15). This indicates that T2DM is a risk factor for developing mild cognitive impairment. The MoCA scores were significantly different between the DR group and the HC group, the T2DM group and the HC group, and the DR group and the T2DM group, which may suggest differences in cognitive function among the three groups, which in turn may prompt diagnosis and early warning. Early clinical support in terms of dysfunction should be provided.

T2DM can play a significant role in the occurrence and/or development of AD either directly or as a cofactor (16). Clinical manifestations such as white matter lesions, cognitive dysfunction, etc., represent the various types of diabetic brain injury (17). Studies have shown that T2DM can cause cognitive decline through changes in hippocampal formation, neurophysiological activity and neurotransmitters (18), and hippocampal formation is one of the first brain structures to be altered (19). Its possible pathological mechanisms are similar to AD at the molecular level, including insulin resistance, metabolic mechanism damage, beta-amyloid (Aβ) formation, oxidative stress, and the presence of advanced glycation end products (AGEs), neuronal apoptosis. van Eldren et al. (19) and most scholars believe that the most important pathological feature of AD is the activation of astrocytes induced by Aβ deposition, which triggers the associated inflammatory response and oxidative stress (17). Although the damage of these nervous systems can be manifested by a variety of examination methods, MRS is uniquely and non-invasively embodied by its unique imaging method, that is, the specific and sensitive expression of the neuronal marker NAA (13). The results of this study showed that the NAA value of the bilateral hippocampal DR group was lower than that of the T2DM group and the HC group, and the NAA value of the bilateral hippocampal T2DM group was lower than that of the HC group (*P* < 0.05). Similar to the results of some studies (20, 21), the above changes suggest that DM can cause neurological damage. Studies have confirmed that NAA reduction is associated with neuronal or axonal loss (22) and is independently associated with the development of T2DM (23). Increased anaerobic glycolysis in the brain is accompanied by elevated blood sugar. When the accumulation of lactic acid increases, acidosis can occur, which in turn destroys the blood-brain barrier. The damage of nerve cells and glial cells is also aggravated by brain edema caused by the process (24). In addition, mitochondrial damage and cell death, DNA fragment breaks and increased cerebral ischemic damage are also caused by persistently elevated hyperglycemia, the mechanism of which is associated with excessive Ca^2+^ channel opening (21). Since Cr is a marker of creatine phosphate, it plays a buffer role in energy metabolism, and its changes in the body are relatively stable. Therefore, the main influences of NAA and NAA/Cr are NAA, and NAA is less sensitive and direct, but it may be weaker than NAA/Cr. There were no statistical differences in the rest of the indicators in this study, similar to previous studies (25).

The results of this study indicate that NAA/Cr, which represents neurons in DR patients, is significantly associated with MoCA scores that reflect cognitive function. Some studies found that there is consistency between the change of MoCA score and ^1^H-MRS, which is partially consistent with the results we obtained (26, 27). It was shown that in the DR patient group, there was synaptic functional remodeling in the hippocampus, which was confirmed in an animal model (28). T2DM spatial learning memory defects (29) are also related. In this study, it was found that anaerobic glycolysis caused by hyperglycemia and excessive opening of Ca^2+^ channels may damage the blood-brain barrier and cause damage to nerve cells and glial cells (30). This damaging change may be specific to NAA or NAA/Cr. In addition, there is insulin receptor resistance in the left hippocampus (31), and the hippocampus is the pancreatic exocrine structure of human insulin, which can produce a small amount of insulin. As an important neuro-influence factor, insulin maintains nerve function, and nerve cell structure. It has an important role in integrity and post-injury regeneration, which means that the damage and degeneration of the left hippocampal neurons in this study are particularly significant (20). There were no statistical differences in the rest of the indicators in this study, similar to previous studies (30). In summary, NAA/Cr can be considered as a biochemical indicator for evaluating cognitive function.

This study shows that DR was the main risk factor for DCI, similar to the results of the study (31). Some Chinese scholars believe that cognitive dysfunction in patients with type 2 diabetes is associated with diabetic microvascular complications (32). Studies have pointed out that in the early stage of diabetic retinopathy, the pathological changes of the nerve fiber layer precede the retinal microangiopathy (33). The mechanism is that the swelling of the nerve cells causes compression of the surrounding microvessels and stenosis (34). Diabetic microvascular disease caused by diabetes may also cause abnormalities in the metabolism of retinal neurons and glial cells, which may cause degeneration of retinal nerve tissue (35). The typical manifestation is a decrease in retinal ganglion cells and a thinning of the retinal nerve fiber layer (34), causing changes in vision. T2DM microangiopathy is very common in patients with diabetic cerebrovascular disease, mainly caused by changes in brain microvascular structure, which increases arteriovenous short circuiting, resulting in reduced transfer of essential nutrients to nerve tissue, insufficient nutrition, decreased perfusion pressure, and reduced cerebral blood (36, 37). It makes brain tissue more susceptible to hypoxic damage. DR with microvascular disease and brain with cognitive decline have many similarities in microvascular structures, such as capillary basement membrane thickening, lumen narrowing, and increased vascular permeability. Both DCI and DR belong to diabetic microangiopathy. Due to their common pathogenesis: AGEs, activation of polyol pathway, oxidative stress, PKC, inflammatory factors, hemodynamic changes, etc., the occurrence and development of both Parallelism, but due to the genetic susceptibility of the two, the difference in the involvement of cytokines, some differences in diagnosis, etc., the non-parallelism of the two, so the severity of DR and DCI have a certain correlation, but not completely related (36, 37).The 5-year incidence of DR is 8%, and the 10-year incidence is 43%; although, DCI is usually seen in diabetic patients for more than 10 years, the incidence of both increases with the duration of diabetes (36, 37). DR and DCI are not only diabetic microangiopathy but also neuropathy, which is sensitive to hypoxia, ischemia, and metabolic disorders. Therefore, diabetic patients may suffer from both diseases after a period of illness. The difference is that the optic nerve, as part of the peripheral nervous system, is relatively more sensitive to hyperglycemia, leading to abnormal hemodynamics, impaired optic nerve nutrient metabolism, and possibly DR earlier than DCI. In addition to the similar pathological mechanisms of DR, DCI also has a large amount of amyloid deposits, which occurs slightly later than DR, but overall, the longer the duration of diabetes, the greater the likelihood of DR and DCI.

### Limitation

DR and DCI with the regularity and correlation of T2DM specific disease course changes, we will improve the group expansion study in the next step.

## Conclusion

Diabetic cognitive impairment is closely correlated with the DR in patients with T2DM. Hippocampal brain metabolism may have some changes in two sides of NAA in patients with DR, and ^1^H-MRS may provide effective imaging strategies and methods for the early diagnosis of brain damage and quantitative assessment of cognitive function in T2DM.

## Data Availability Statement

All datasets generated for this study are included in the manuscript/supplementary files.

## Ethics Statement

This study was carried out in accordance with the recommendations of the Declaration of Helsinki, Renmin Hospital of Wuhan University Clinical Research Ethics Committee. The protocol was approved by the Renmin Hospital of Wuhan University Clinical Research Ethics Committee. All subjects gave written informed consent in accordance with the Declaration of Helsinki.

## Author Contributions

All authors listed have made a substantial, direct and intellectual contribution to the work, and approved it for publication.

### Conflict of Interest

The authors declare that the research was conducted in the absence of any commercial or financial relationships that could be construed as a potential conflict of interest.

## Abbreviations

T2DM: Type 2 diabetes mellitus
DCI: diabetic cognitive impairment
DR: diabetic retinopathy
^1^H-MRS: proton magnetic resonance spectroscopy
HC group: healthy control group
MoCA: Montreal Cognitive Assessment
NAA: Nacetylaspartate
Cr: Creatine
Cho: Cho-line
mI: myo-inositol
ANOVA: Analysis of Variance.
